# Biochemical characterization of recombinant influenza A polymerase heterotrimer complex: Polymerase activity and mechanisms of action of nucleotide analogs

**DOI:** 10.1371/journal.pone.0185998

**Published:** 2017-10-11

**Authors:** Ona Barauskas, Weimei Xing, Esmeralda Aguayo, Madeleine Willkom, Annapurna Sapre, Michael Clarke, Gabriel Birkus, Brian E. Schultz, Roman Sakowicz, HyockJoo Kwon, Joy Y. Feng

**Affiliations:** Gilead Sciences, Inc., Foster City, California, United States of America; Universidad Autonoma de Madrid Centro de Biologia Molecular Severo Ochoa, SPAIN

## Abstract

Influenza polymerase is a heterotrimer protein with both endonuclease and RNA-dependent RNA polymerase (RdRp) activity. It plays a critical role in viral RNA replication and transcription and has been targeted for antiviral drug development. In this study, we characterized the activity of recombinant RdRp purified at 1:1:1 ratio in both ApG-primed RNA replication and mRNA-initiated RNA transcription. The heterotrimer complex showed comparable activity profiles to that of viral particle derived crude replication complex, and in contrast to the crude replication complex, was suitable for detailed mechanistic studies of nucleotide incorporation. The recombinant RdRp was further used to examine distinct modes of inhibition observed with five different nucleotide analog inhibitors, and the apparent steady-state binding affinity *K*_*app*_ was measured for selected analogs to correlate antiviral activity and enzymatic inhibition with substrate efficiency.

## Introduction

Influenza A viruses cause recurrent epidemics and global pandemics that claim the lives of millions. The emergence of novel strains and variants will continue to pose challenges to public health [1]. Influenza viruses belong to the family of Orthomyxoviridae viruses and are classified into 16 HA subtypes (H1-H16) and 9 NA subtypes (N1-N9) based on the antigenicity of their hemagglutinin (HA) and neuraminidase (NA) proteins [1, 2]. Like all members of this family, the viral genome is composed of eight segments of single-strand, negative-sense RNA that encode 10 viral proteins: three proteins that form viral RNA-dependent RNA polymerase (RdRp) (PA, PB1 and PB2), four structural proteins (HA, NA, M1 matrix protein and the M2 ion channel protein), two nonstructural proteins (NS1A and NS2/NEP), and a nucleoprotein (NP) [2, 3]. Multiple antiviral drugs targeting various stages of the viral life cycle have been approved or are in clinical trials for treatments of influenza [4–6].

Influenza A virus ribonucleoprotein complexes (RNPs) are responsible for viral RNA transcription and replication and are central to the viral life cycle [7]. The RNA comprises a linear viral genome, a single RdRp, and multiple copies of NP monomers. The RdRp is formed by the association of acidic subunit PA and two basic subunits PB1 and PB2. The N-terminal domain of PA functions as an endonuclease, while its C-terminal domain interacts with PB1 [8–10]. The PB1 subunit performs both de novo RNA-dependent RNA synthesis and mRNA-primed transcription. PB1 contains four conserved motifs that form a large catalytic domain at the center of the protein [11, 12]. The N-terminal domain of PB1 interacts with PA, while its C-terminus interacts with the N-terminus of PB2 [13], forming the RdRp heterotrimer complex. PB2 binds the m^7^guanosine (m^7^G) cap of host pre-mRNAs and enables downstream cleavage by the endonuclease domain of PA. Cleavage of host pre-mRNA generates a m^7^G capped10-13-mer oligonucleotide that serves as a primer for viral RNA transcription. Crystal structures have demonstrated that cap-binding domain of PB2 binds a cap analog m^7^GTP while the N-terminal domain interacts with the C-terminus of PB1 [14, 15]. In addition, PB2 plays important roles in polymerase activity, host range, cold sensitivity, and pathogenesis [8, 16]. Nucleotide analog inhibitors targeting the influenza RdRp complex have been an attractive strategy due to frequent occurrence of drug-resistant viruses to M2 ion channel blockers and neuraminidase inhibitors. A clear and detailed mechanism of inhibition of polymerase inhibitors is crucial for the design of effective antivirals.

Nucleoside/nucleotide analogs have played key roles as antiviral agents for herpes simplex virus (HSV), human immunodeficiency virus (HIV), hepatitis B virus (HBV), hepatitis C virus (HCV), and most recently Ebola virus (EBOV) [17, 18] [19]. 2’-deoxy, 2’F guanosine (2’FdG) [20] [21, 22] and 2’-deoxy, 2’F cytidine (2’FdC) [23] showed potent anti-influenza activity in vitro and in vivo models, however neither advanced to the clinic. T-705 (Favipiravir) is the only approved influenza antiviral but is limited for use during pandemics [24, 25]. The discovery of new nucleoside analogs has been hampered by the difficulty of obtaining recombinant RdRp heterotrimer complex. In recent years, several groups have studied recombinant RdRp either in the form of partially purified protein, with PA subunit in significant excess, or using chimeric complexes with subunits expressed from different viral strains [26–30].

We recently reported the expression and purification of a homogeneous trimeric polymerase complex[31]. In this study, we have characterized the replication and transcription activities of the recombinant polymerase complex and used it to determine distinct modes of action for five nucleoside inhibitors of influenza virus. The recombinant polymerase complex and defined templates allowed visualization of single nucleotide incorporation of the active metabolite of Favipirivir and related analog T-1106 triphosphate, which were not direct chain terminators and could not be observed in RNP or crude polymerase reactions. Further engineering of template sequences elucidated unique modes of action of Favipiravir and related analog T-1106 triphosphate, as well as 2’FdG and two additional modified guanosine analogs. In addition to mode of action, the recombinant polymerase complex was used to determine relative incorporation efficiency, one of the measures of intrinsic potency for nucleotide antivirals.

## Materials and methods

### Reagents

MDCK (NBL-2) cells were obtained from ATCC (Manassas, VA) and cultured in Dulbecco's modified Eagle's medium (DMEM) from Life Technologies (Carlsbad, CA) supplemented with 10% heat inactivated fetal bovine serum (Hyclone, Logan, Utah) and 100U/ml penicillin-streptomycin (Life Technologies, Carlsbad, CA). Influenza A viruses A/Hong Kong/8/68 and A/Puerto Rico/8/34 were purchased from Advanced Biotechnology Inc. (Columbia, MD).

**Fig 1:**
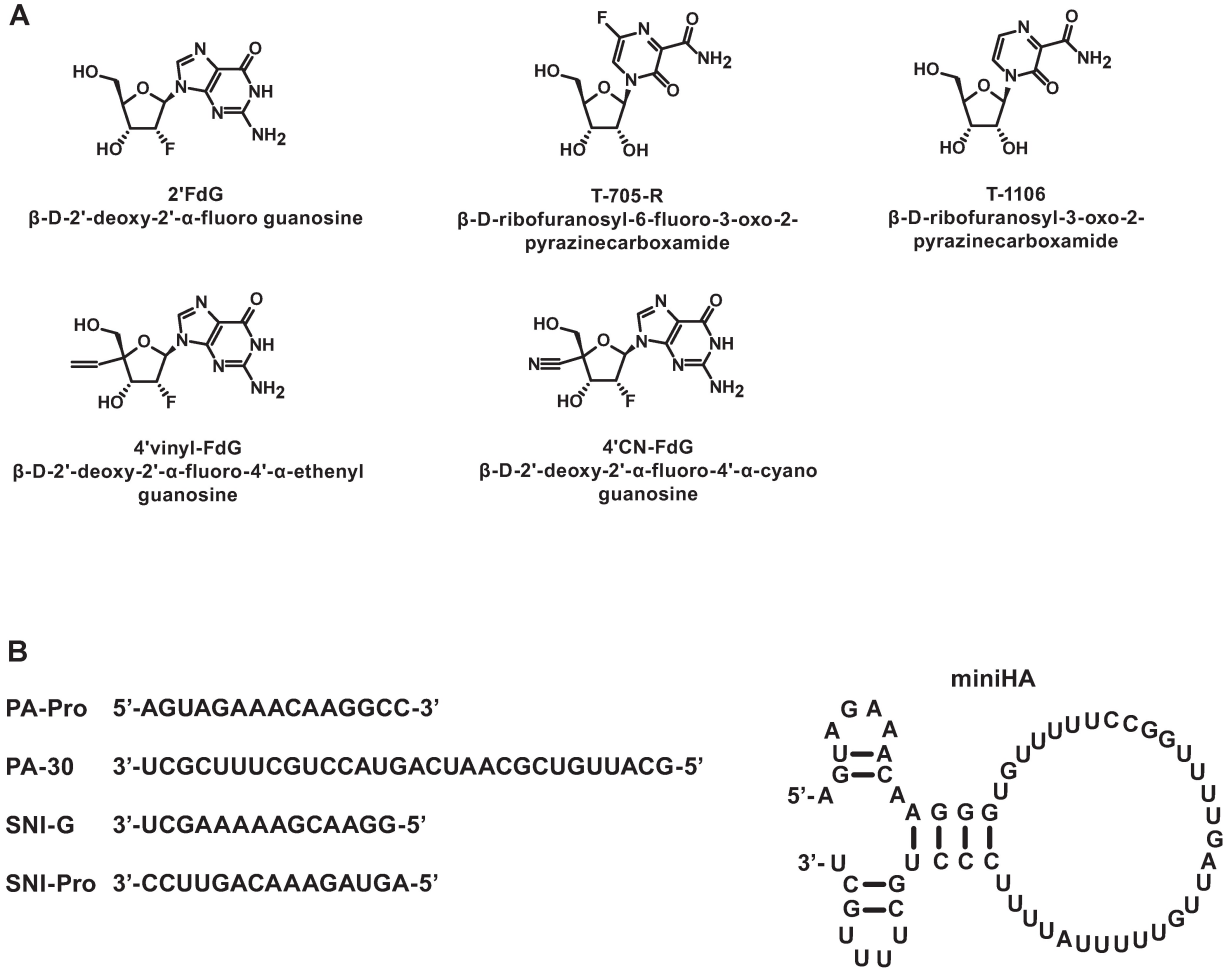
Chemical structures of nucleotide analogs (A) and RNA oligonucleotides (B) used in this study.

All natural nucleotide triphosphates ATP, GTP, UTP, and CTP were obtained from GE Healthcare (Pittsburgh, PA). Radiolabeled nucleotides α-^33^P-GTP, UTP, and ATP (3000 Ci/mmol, 10 mCi/mL) were purchased from PerkinElmer (Waltham, MA). All of the nucleoside analogs shown in Fig 1A and their triphosphate (TP) forms were synthesized at Gilead Sciences except 2’FdG for which TP was from TriLink Biotechnologies (San Diego, CA). All synthetic RNA oligonucleotides (Fig 1B) were synthesized by TriLink Biotechnologies. These include PA-30, a 30-mer template analogous to the 3’ end of the PA gene; PA-Pro, a 15-mer promoter with partially complementary sequence to PA-30, SNI-G, a template for single nucleotide incorporation (SNI) with GTP or GTP analog; SNI-Pro, the corresponding promoter oligo used in SNI studies as described by Jin et al. [26]; and the miniHA template resembling the 3’- and 5’- ends of the HA gene with the panhandle promoter structure [32] as illustrated in Fig 1B.

The radiolabeled cap-1 mRNA primer ^33^P-m^7^G^1^67 was prepared as described previously [20]. Briefly, 10 μg pGEM zf(+) plasmid was digested with 800 U SmaI at room temperature overnight and purified by ethanol precipitation. RNA transcription was carried out at 37°C overnight using the MegaScript Sp6 transcription kit (Invitrogen, Carlsbad, CA) according to the manufacturer’s protocol. After 10 minute incubation with DNAse I, the final RNA was recovered by phenol chloroform extraction. 5’-capping was achieved by the following: 60 μg of RNA transcript was denatured by heating at 95°C for 10 minutes, cooled to room temperature and added to a capping reaction mixture containing the ScriptCap m^7^G Kit, 2’-O-methyl transferase enzyme (CellScript, Madison, WI), and 500 μCi of α-^33^P-GTP for 6 hours at 37°C in a final volume of 100 μL. The final cap-1 RNA product was inactivated at 95°C for 10 minutes and passed through G25 spin columns (GE Healthcare, Pittsburgh, PA) to remove unincorporated nucleotide.

### Baculovirus expression and purification of recombinant influenza A RNA polymerase

The PA, PB1 and PB2 genes of Influenza A virus RNA polymerase were amplified by reverse-transcription and PCR from H1N1 strain A/PR/8. The 3x FLAG tag was added to the N-terminal full-length PA and cloned into pFastBac1 vector (Life Technologies, Carlsbad, CA). Full-length PB1 and PB2 genes were cloned into pFastBac Dual vector (Life Technologies) as described previously[31]. Recombinant baculovirus was generated for PA and PB1/PB2 using Sf9 cells and was used to co-infect *Trichopulsia ni* cells (Hi5) at 1.5 x 10^6^ cells/ml in ESF-921 medium (Expression Systems, David, CA). Purification and biochemical characterization of the polymerase heterotrimer was described previously [31]. The purified polymerase complex was stored at -80°C prior use.

### Isolation of crude ribonucleoprotein complex

Crude ribonucleoprotein (cRNP) complex was prepared from virions by detergent disruption as described previously [25, 33]. Briefly, 3 mg influenza A/PR/8/34 (H1N1) viral stock (~1.8 x 10^12^ viral particles, Advanced Biotechnologies Inc., Eldersburg, MD) was pelleted at 54,000g for 2 hours. The pellet was disrupted by exposure to 2% Triton X-100 for 30 minutes at room temperature in a total of 100 μL buffer containing 100 mM Tris-HCl (pH 8.0), 200 mM KCl, 3 mM dithiothreitol (DTT), 10% glycerol, 10 mM MgCl_2_, 2 U/mL RNasin Ribonuclease Inhibitor (Promega, Madison, WI), and 2 mg/mL Lysolechithin type V (Sigma-Aldrich, St. Louis, MO). Aliquots of cRNP were stored at -80°C.

### Activity against influenza A virus (A/Hong Kong)

MDCK cells were seeded in 96-well plates at a density of 1 × 10^4^ cells per well in 100 μL of MEM medium supplemented with 10% FBS. Medium was removed the next day and the cells were washed twice with 100 μL MEM medium supplemented with 0.125% (w/v) BSA (MEM-BSA). Serially diluted compounds were added to cells in 150 μL MEM-BSA and cells were infected with influenza A (A/Hong Kong/8/68, Advanced Biotechnologies Inc.) at MOI 0.005 in 50 μL of MEM-BSA medium supplemented with 8 μg/mL trypsin (Worthington, Lakewood, NJ). After five day incubation at 37°C, virus-induced cytopathic effect was determined by adding CellTiterGlo viability reagents (Promega, Madison, WI) and the luminescence signal was measured using Perkin Elmer’s Envision MultiLabel plate reader (Santa Clara, CA). The cytotoxicity of compounds in MDCK cells was determined in replicate plates with the same setup as used in the antiviral assay, except no virus was added to the cell culture. EC_50_ and CC_50_ values were calculated by non-linear regression analysis using GraphPad Prism (La Jolla, CA) and four parameter logistic sigmoidal dose-response (variable slope) equations.

### Influenza polymerase inhibition (IC_50_) assays

Nucleotide analog inhibitors were serially diluted 3-fold in water and added to reaction mixture containing 10% virus cRNP (v/v), 100 mM Tris-HCl (pH 8.0), 100 mM KCl, 1 mM DTT, 10% glycerol, 0.25% Triton-101 (reduced), 5 mM MgCl_2_, 0.4 U/mL RNasin, and 200 μM ApG dinucleotide primer (TriLink, San Diego, CA). Reactions were initiated by addition of ribonucleotide triphosphate (NTP) substrate mix containing 1 μM GTP (at 0.5 x *K*_m_), 0.01 μM α-^33^P GTP, and 100 μM of ATP, CTP, and UTP (PerkinElmer, Shelton, CT). Reactions were incubated at 30°C for 90 minutes then spotted onto DE81 filter paper. Filters were air dried, washed with 0.125 M Na_2_HPO4 (3×), water (1×), and Ethanol (1×) and air dried before exposure to Typhoon phosphor imager screen. Product formation was quantified on a Typhoon Trio (GE Healthcare, Piscataway, NJ). IC_50_ values were calculated for inhibitors by fitting the data to a four-parameter sigmoidal dose response with variable slope in GraphPad Prism.

### Influenza RdRp enzyme activity assays

The polymerase activity of RdRp was studied in a similar manner as in the IC_50_ assay described above. The recombinant polymerase trimer was pre-incubated for 5 minutes in a buffer containing 50 mM Tris-HCl (pH 8.0), 2 mM DTT, 5 mM magnesium acetate, 0.25 U/μL RNAsin, 1.6 μM PA-30 template, 1.6 μM PA-Pro promoter or miniHA template and one of the two RNA primers: 300 μM ApG dinucleotide primer (TriLink, San Diego, CA) for replication activity, or 13 ng/μL rabbit globin mRNA (Sigma-Aldrich) for transcription. Reactions were initiated by addition of NTP substrate mixture containing 0.01 μM α-^33^P-GTP,1 μM GTP, and 100 μM for each of the rest of NTPs: ATP, CTP and UTP (PerkinElmer, Shelton, CT). For the activity assays, reactions were incubated at 30°C and 5 μL aliquots were spotted onto DE81 filter paper over a 60-minute time course. The filters were processed as mentioned above and exposed to a phosphor imager and the amount of α-^33^P-GTP was quantified on a Typhoon Trio (GE Healthcare, Piscataway NJ). Unwashed filters containing known quantities of radiolabel were used to calculate rates of incorporation for ^33^P-GTP. To visualize products, aliquots of the reactions were quenched with equal volumes of gel loading dye containing 90% formamide, 100 mM EDTA, 0.1% (w/v) bromphenol blue and xylene cyanol. Reactions were analyzed by electrophoresis (10% polyacrylamide, 8 M urea). The dried gels were exposed to phosphorimager screen and visualized using the Typhoon Trio and ImageQuant Software (GE, Piscataway, NJ.)

### Single nucleotide incorporation assay using radiolabeled 5’-capped mRNA primer

Recombinant influenza polymerase trimer (80 ng/μL) was incubated with 595 nM ^33^P-m^7^G^1^67 and 1.6 μM miniHA template in buffer containing 50 mM HEPES (pH 7.8), 2 mM DTT, 5 mM magnesium acetate, 0.2 U/mL RNasin for 60 minutes at 37°C. After the endonuclease cleavage of ^33^P-m^7^G^1^67 was completed, 20 μM of endonuclease inhibitor 4-(1,4-bis(4-chlorobenzyl)piperidin-4-yl)-2,4-dioxobutanoic acid [34] was added to the reaction mixture. The polymerase reactions were initiated by adding nucleotide analogs and three other natural NTPs (all at final concentrations of 500 μM) and 10 μCi α-^33^P-ATP. Reactions were incubated at 37°C for 30 minutes and quenched with equal volumes of gel loading dye containing 90% formamide, 100 mM EDTA, 0.1% (w/v) bromphenol blue and xylene cyanol. Reactions were analyzed by electrophoresis (25% polyacrylamide, 8 M urea). The dried gels were exposed to phosphorimager screen and visualized using the Typhoon Trio and ImageQuant Software (GE, Piscataway, NJ.)

### Determination of the apparent binding affinity of NTP analogs

The apparent binding affinity *K*_*app*_ was determined for GTP and T-1106-TP as described by Jin et al [26]. 250 nM recombinant polymerase trimer was pre-incubated with 2.5 μM SNI-G template, 1 μM SNI-Pro promoter, and 400 μM ApG primer in a buffer containing 40 mM Tris-HCl (pH 7.0), 20 mM NaCl, 5 mM MgCl_2_, 2 mM DTT, and 0.2 U/μL RNasin at 37°C. The reaction was initiated by adding a mixture of 25 μM CTP, 25 μM UTP, 25 μCi of α-^33^P-UTP, and various concentrations of GTP or analogs. Reactions proceeded for 35 minutes and were quenched with equal volumes of loading dye containing 90% formamide, 100 mM EDTA, 0.1% (w/v) amount of bromphenol blue and xylene cyanol. The products were separated by 25% PAGE, visualized, and quantified by autoradiography as described above. Data were fit with Eq I, where *%Max* and *%Min* are respectively the ratios of (10+11+12+13+14-mer)/ (9+10+11+12+13+14-mer) at the highest nucleotide concentration and the baseline level of mis-incorporation of CTP/UTP in the absence of the correct nucleotide (GTP) encoded by the template sequence, *K*_*app*_ is the concentration at which half of the reactant (9-mer) is converted to products (10+11+12+13+14-mers), and *S* is the concentration of nucleotide analog.

$$Y=\frac{\left(\%Max-\%Min\right)\cdot S}{K_{app}+S}+\%Min$$ (I)

## Results

### RNA replication and transcription monitored by filter binding assay

The polymerase activity of the purified RdRp heterotrimer complex was characterized in a filter binding assay utilizing the RNA template PA-30, a 30-mer containing a conserved 12 nucleotide sequence from the 3’-end of the Influenza A RNA encoding the PA protein, and a partially complementary 15-mer promoter sequence mimicking the 5’end of the viral genome, PA-Pro (Fig 1). A dinucleotide ApG primer was used to investigate the replication step, while rabbit globin mRNA was used to study mRNA-primed transcription. Recombinant RdRp with exogenous template was assayed in parallel with cRNP from detergent disrupted viral particles containing endogenous viral RNA templates. The incorporation of radiolabeled GTP was used to measure the product formation.

**Fig 2:**
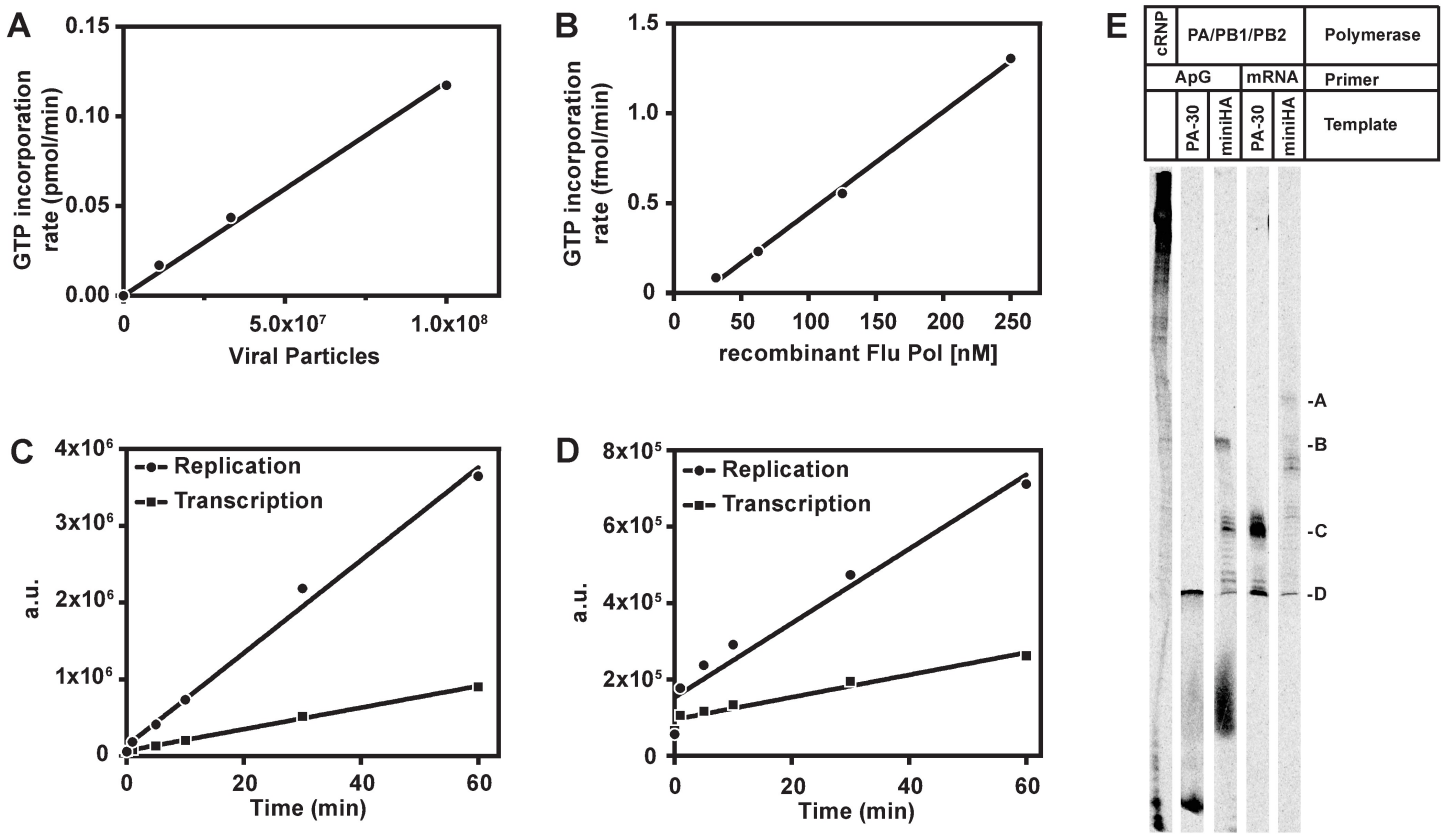
Activity of RNA replication and transcription by recombinant influenza RdRp heterotrimer. (A) The rate of ^33^P-α-GTP incorporation by cRNP is proportional to the estimated viral particles used to generate cRNP; (B) The rate of ^33^P-α-GTP incorporation by recombinant RdRp is proportional to the PA/PB1/PB2 trimer protein concentration; (C and D) The product formation presented by an arbitrary unit on the phosphor imager is linear with time and similar between the replication and transcription rates observed with cRNP (panel C) and recombinant RdRp (panel D) during ApG-primed RNA replication (●) or rabbit globin mRNA-primed RNA transcription (■). (E) Product formation using different RNA templates was analyzed on a 10% polyacrylamide gel. In contract to cRNP-catalyzed RNA synthesis (far left lane) which generated a smeared ladder of long products at the top of the gel, recombinant RdRp generated approximately template length products during ApG-primed RNA replication (D, B). Furthermore, transcripts of PA-30 and mini-HA containing an additional 9–11 nucleotides were generated during rabbit globin mRNA-primed RNA transcription (C, A).

A comparison of the enzymatic activities of cRNP and recombinant RdRp is shown in Fig 2. 2A shows GTP incorporation rate by crude replication complex as a function of viral particles, and 2B shows incorporation rates as a function of recombinant polymerase concentration using the PA-30 template. Although the templates were different for the recombinant RdRp and the cRNP, the relative replication and transcription activities were comparable between these two sources of polymerase. Replication and transcription activities of cRNP and the recombinant polymerase were linear over time (Fig 2C and 2D), and neither recombinant RdRp nor cRNP showed de novo RNA synthesis in the absence of a primer (S3 Fig). This allowed for analysis of the two distinct modes of RNA synthesis: ApG-primed RNA replication or capped mRNA-primed RNA transcription.

### RNA replication and transcription monitored by gel electrophoresis

The polymerase activity of RdRp heterotrimer complex was further characterized in parallel with that of the cRNP in a gel-based assay in which the RNA products could be visualized. No exogenous RNA template was provided for cRNP whereas two RNA templates, PA-30 and miniHA, were used with RdRp. Results are shown in Fig 2E. Shown in lane 1, cRNP generated long transcripts near the top of the gel and short abortive initiation products. Lane 2 shows a transcript of the PA-30 template generated by the recombinant trimer and short abortive initiation products. Similarly, the recombinant trimer generated transcripts corresponding to the miniHA template, as well as intermediate length transcripts and abortive initiation products (lane 3). In mRNA primed reactions with recombinant trimer (lanes 4 and 5), abortive initiation was not observed and the products are correspondingly ~9–11 nucleotides longer than the PA-30 template (lane5) and miniHA template (lane 6). MiniHA has been predicted to form a double hairpin ‘panhandle’ promoter structure by mimicking the viral genome and thus enhancing RdRp activity[35], while PA-30 transcription is increased with a semi-complementary promoter sequence mimicking the 5’-end of viral RNA described previously[27].

### Characterization of nucleotide analogues in Influenza polymerase inhibition (IC_50_) assays and antiviral assays (EC_50_)

Five guanosine nucleotide analogs (Fig 1) were tested for inhibition of ApG-primed RNA synthesis catalyzed by cRNP complex in filter binding assays. The longer endogenous viral template and low polymerase concentration in cRNP afforded better sensitivity than the recombinant polymerase trimer for IC_50_ measurements. Antiviral activity was measured in cell-based viral replication assays. Potency and cytotoxicity data from these assays are shown in Table 1. The biochemical potency of the triphosphates ranged from 0.17 μM to 80 μM, in the order of T-1106-TP > T-705-RTP > 2F’dGTP > 4’vinyl-FdGTP > 4’CN-FdGTP. In the cellular anti-viral assays, T-705 showed the highest activity with EC_50_ of 2.2 μM, followed by 2’FdG, T-1106, and 4’vinyl-FdG with EC_50_ values of 18, 27 μM, and 110 μM, respectively, while 4’CN-FdG remained inactive at the highest concentration tested (200 μM). The rank ordering of compounds in the biochemical assay paralleled that of the cellular assays, with the exception of T-1106-TP. This compound was 3-times more potent than T-705-RTP in the biochemical assay, but was 12-times less potent in the antiviral assays. We speculate that this discrepancy may be caused by insufficient formation of active triphosphate of T-1106 in the MDCK cells used in the antiviral assay.

**Table 1:** Inhibition of influenza virus and crude replication complex.

	Cell-based		Biochemical
Nucleoside Analogs	EC_50_ (μM)^1^^,^^2^	CC_50_ (μM)	IC_50_ of TP (μM)^1^^,^^3^
2’FdG	19 ± 5	> 200	3.8 ± 2.6
T-705	2.4 ± 1.1	> 100	0.56 ± 0.22
T-1106	27 ± 6	> 100	0.17 ± 0.07
4’vinyl-FdG	116 ± 48	> 200	24.0 ± 0.2
4’CN-FdG	> 200	> 200	80 ± 25

^1^All values are average ± STD from at least three independent measurements.

^2^EC_50_ values were measured in MDCK cells infected with Influenza A/HK/68.

^3^IC_50_ values were determined using viral ribonucleoprotein complexes from detergent-disrupted Influenza A/PR/8/34.

### Mechanism of action for NTP analog inhibitors

**Fig 3:**
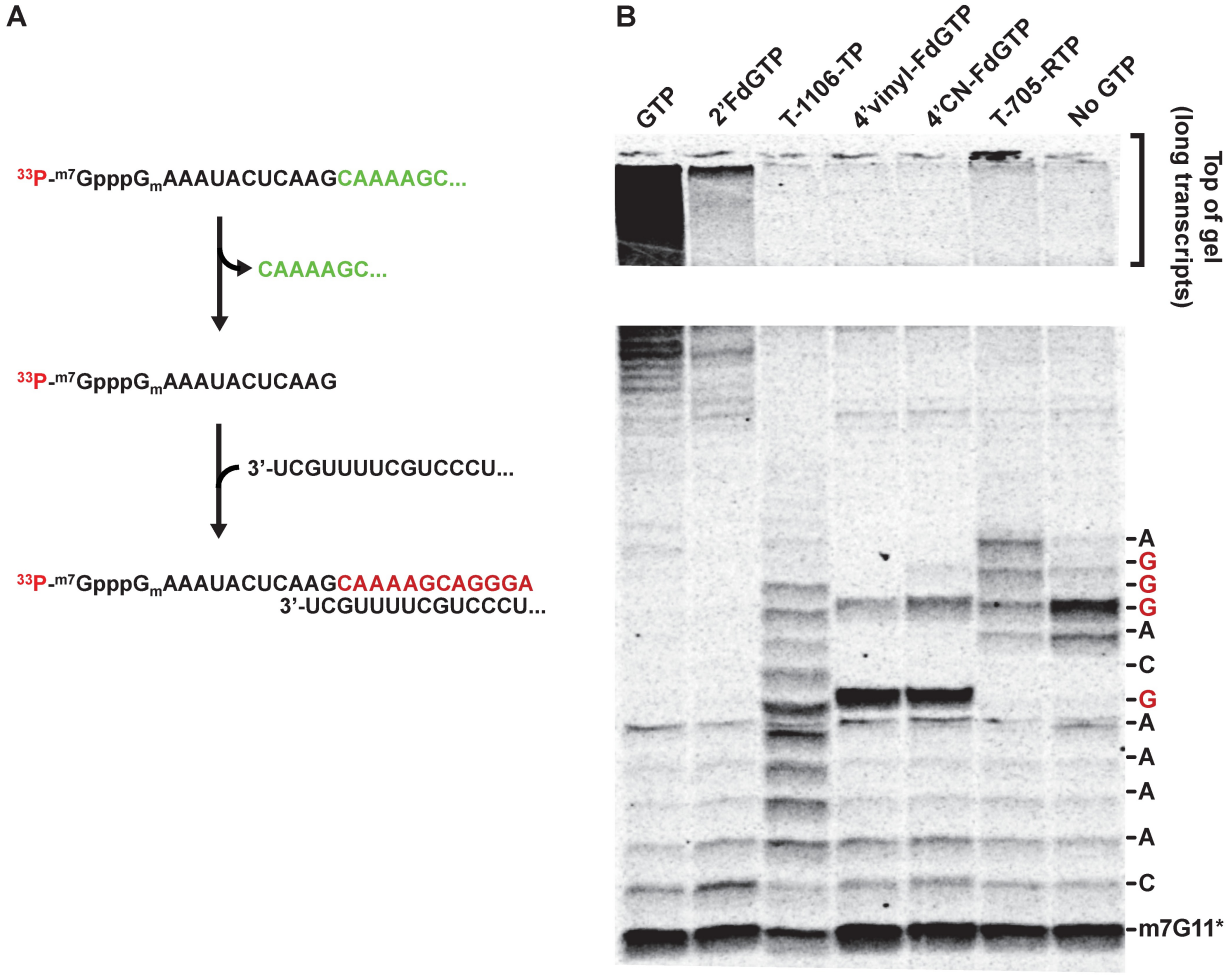
Mechanism of action for different nucleotide analogs during recombinant RdRp-catalyzed RNA synthesis. (A) Reaction scheme showing cleavage of the ^33^P-m^7^G^1^67 primer by influenza polymerase heterotrimer, base pairing with miniHA template, and expected transcription product. (B) Reaction mixtures contained miniHA template, radiolabeled capped RNA primer ^33^P-m^7^G^1^67, 500 μM GTP or GTP analogs, and 500 μM for each of natural CTP, UTP, and ATP. Products were separated by 25% PAGE and visualized by autoradiography. The top of the gel was illustrated to show the formation of long transcription products. The ladder of nucleotide bases on the side bar demonstrates the correct incoming nucleotide base-pairing with the miniHA template.

Development of gel based assays using recombinant polymerase complex and defined templates enabled characterization of mechanisms of action of nucleotide inhibitors, which could not be distinguished using cRNP complex containing viral RNA. The mechanisms of action for five guanosine triphosphate analogs were examined in a gel-based assay using the miniHA template and a capped radiolabeled mRNA m^7^G^1^67. After pre-cleavage of the radiolabeled primer, a specific endonuclease inhibitor 4-(1,4-bis(4-chlorobenzyl)piperidin-4-yl)-2,4-dioxobutanoic acid was added to inhibit endonuclease activity and prevent cleavage of the newly elongated transcripts formed in the presence of NTPs or NTP analogs. As shown in Fig 3, the template sequence allows visualization of multiple guanosine or adenosine analog incorporations. In the reaction containing all four natural NTPs at 500 μM concentration (lane 1), the majority of radiolabeled products accumulated near the top of the gel. When 2’FdGTP was substituted for GTP (lane 2), a reduced amount of radiolabeled RNA was present at the top of the gel; however, the lack of chain-terminated transcripts suggested that the analog was stably incorporated into RNA. T-705-RTP has been reported to be incorporated through base-pairing with either cytosine or uracil by influenza RdRp, terminating RNA synthesis after two consecutive incorporations [26, 36]. Our single nucleotide incorporation data in Fig 3 is in general agreement with these observations. In lane 3, T-1106-TP shows competition with ATP for incorporation opposite U as well as GC base pairing, and delayed chain termination. This blocks formation of long transcripts after multiple incorporations. The 4’-vinyl and 4’-cyano-FdGTP analogs (lanes 4 and 5) showed direct chain-termination at the first guanosine incorporation site and blocked formation of long products. Interestingly, even though T-705-RTP (lane 6) and T-1106-TP are close analogues, T-705-RTP generated a product pattern significantly different from that of T-1106-TP. T-705-RTP decreased competition with adenosine with a clear ladder of different sized products; decreased accumulation of the first GTP incorporation product; and could stably incorporate and increase long product at the top of the gel. In the absence of GTP or its analog (lane 7), the polymerase was able to bypass the first GTP site through mis-incorporation of other NTPs, but transcription is stalled at the second GTP incorporation site and no formation of longer transcripts were observed. The recombinant RdRp heterotrimer enabled the detailed study of these five structurally diverse compounds which was not feasible using cRNP (S2 Fig).

### Steady-state kinetic analysis for single nucleotide base incorporation

**Fig 4:**
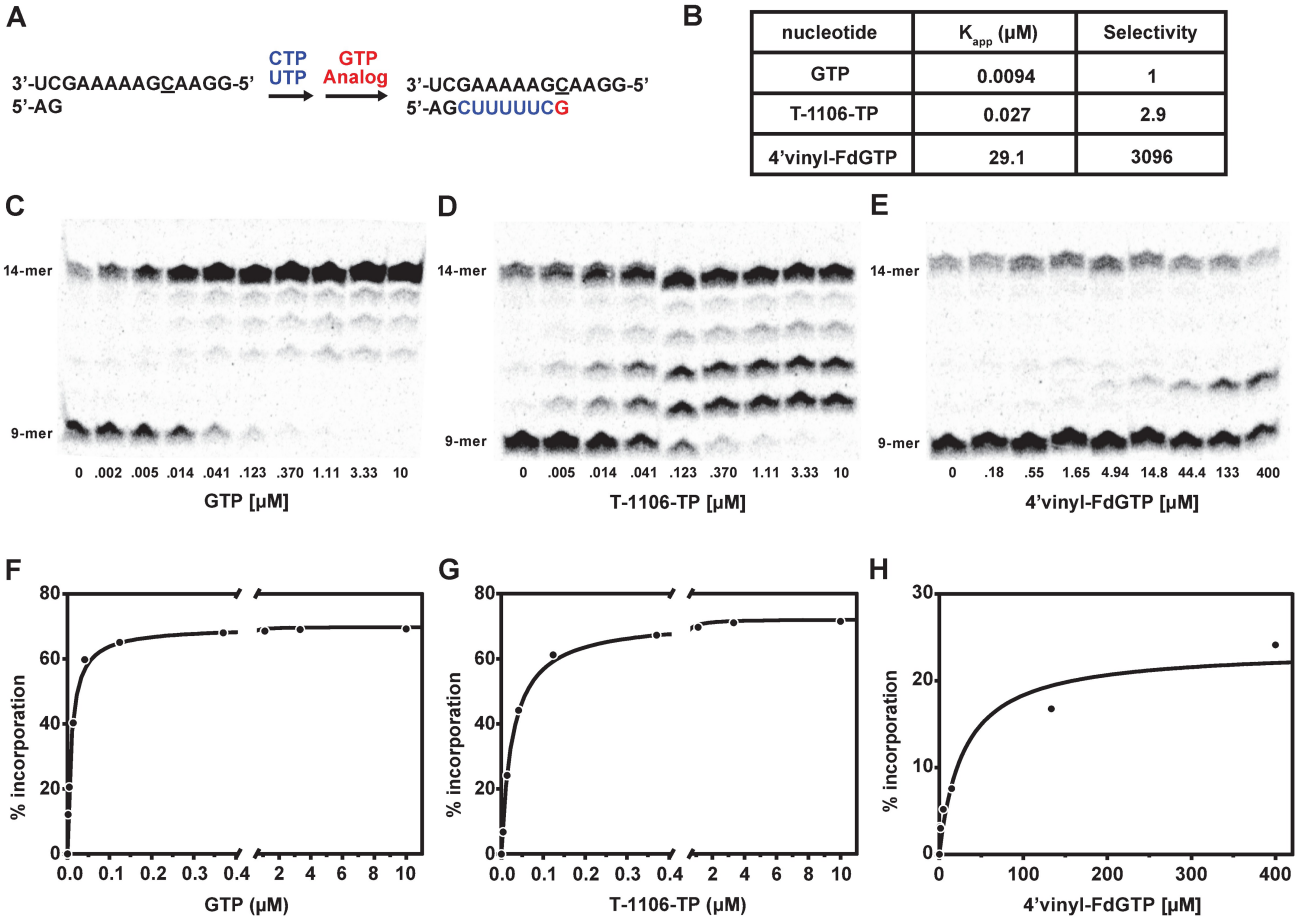
Determination of apparent binding affinity (*K*_app_) of GTP, T-1106-TP, and 4’vinyl-FdGTP during single nucleotide incorporation by the recombinant influenza RdRp. (A) Diagram illustrating formation of RNA 9-mer product from ApG primer and further elongation to RNA 14-mer product; (B) Summary of *K*_app_ and selectivity for GTP and analogs; Incorporation of GTP (panel C), T-1106-TP (panel D), and 4’vinyl-FdGTP (panel E) as a function of NTP analog concentration. The product formation is plotted as a function of compound concentration for GTP (panel F), T-1106-TP (panel G), and 4’vinyl-FdGTP (panel H), respectively. The calculated *K*_*app*_ values for GTP, T-1106-TP, and 4’vinyl-FdGTP are 9 nM, 27 nM, and 29 μM respectively.

The recombinant polymerase assay system also enabled comparison of substrate incorporation efficiency between natural substrates and nucleotide analogs. We studied single nucleotide incorporation for three GTP analogs with a pre-formed RdRp-RNA primer/template elongation complex. While we were unable to define pre-steady state kinetic parameters *k*_*pol*_ and *K*_*d*_ due to the slow, highly abortive initiation and mis-incorporation of natural nucleotides, we measured the concentration of nucleotide analog required to elongate 50% of a RNA 9-mer to 10-14-mer products in a fixed short reaction time (*K*_*app*_), which provides a relative comparison of substrate efficiency. The *K*_app_ values for GTP, T-1106-TP, and 4’-vinyl-FdG are summarized in Fig 4B. The ratio of *K*_app(analog)_:*K*_app(GTP)_ provides a selectivity factor. The *K*_*app*_ for GTP was calculated to be 9 nM, consistent with the reported value of 8 nM [26]. The *K*_*app*_ value of 27 nM for T-1106-TP yields a selectivity factor of 3 for natural GTP over T-1106-TP, 6.3 times lower than the reported selectivity over T-705-TP [26]. These data were consistent with the 3-fold lower enzymatic IC_50_ observed for T-1106-TP vs. T-705-RTP (Table 1). *K*_*app*_ for 4’vinyl-FdGTP was 29 μM, in agreement with weak enzymatic inhibition observed with this analog.

## Discussion

Xing, et al recently described the purification of the recombinant influenza polymerase heterotrimer and showed that the endonuclease activity was significantly increased over the broadly used N-terminal truncated PA subunit[31]. In this paper, we evaluated the RdRp activity of this heterotrimer complex during both ApG-primed RNA replication and mRNA-dependent RNA transcription. Similar to the viral-derived cRNP, the recombinant RdRp appears to be 2-fold more efficient during replication than transcription, indicating endonuclease cleavage could be rate-limiting during mRNA transcription. Using the recombinant RdRp and sequence-defined RNA template and primer, we were able to investigate the mechanism of action for nucleoside analogs active against influenza virus. Most of the marketed nucleoside antivirals exert their effects by incorporation into nascent DNA or RNA and subsequent chain termination. However, nucleoside inhibitors of influenza virus demonstrated distinct modes of action. The stable incorporation of 2’FdGTP suggested a kinetic inhibition mechanism of action, in which the nucleoside analog disrupts and delays extension of RNA. However, this observation was in direct conflict with Tisdale’s report of direct chain-termination upon 2’FdGTP incorporation [20]. We have observed similar delayed chain-termination effect of T-705RTP as reported by Jin et al [26]. The fact that T-705RTP and its close analog T-1106-TP can be incorporated as either “A or G” bases also suggests hypermutation as an antiviral mechanism, described by Crotty et al as accumulation of mutations in viral RNA leading to a drop in viral fitness[37]. While these two analogs cause chain termination after two or more subsequent incorporations, the likelihood of more than two subsequent incorporations of these analogs in the presence of physiological levels of natural NTP is small. The increased potency of T-1106-TP with respect to T-705-RTP correlates with its enhanced ability to be incorporated as adenosine (Fig 3). Interestingly, both 4’vinyl- and 4’CN-FdGTP analogs showed a clear chain-termination effect. Understanding modes of action of nucleotide inhibitors may identify potential efficacy or toxicity liabilities and guide drug development. Using crude replication complex we were unable to observe incorporation of T705-RTP or the 4’vinyl and 4’CN-FdGTP analogs, suggesting that they may not be substrates of influenza polymerase (S2 Fig). The recombinant polymerase complex and defined templates also demonstrated reduced mis-incorporation of natural nucleotides, allowing distinction between stably incorporated analogs such as 2’FdGTP and the chain terminators 4’vinyl- and 4’CN-FdGTP. The 4’vinyl- and 4’CN-FdGTP did not block formation of long transcripts in cRNP assays due to poor substrate efficiency combined with significant mis-incorporation of natural nucleotides. Recombinant influenza A polymerase heterotrimer provided a useful tool to elucidate subtle mechanisms of action differences for nucleoside antivirals. By using highly purified recombinant influenza A polymerase heterotrimer and defined RNA templates, we have shown how five different nucleotide analogs with seemingly subtle structure modifications could lead to very different mechanisms of action. These findings will enable future efforts to develop more effective and selective nucleoside antivirals targeting influenza polymerase.

## Supporting information

S1 Fig Methylation of capped primer. Methylation of m^7^G_0_-67mer RNA was assessed by incorporation of ^14^C from trace labeled S-adenosyl methionine (SAM), (PerkinElmer, Walthham, MA) using the ScriptCap vaccinia virus 2‘-O-Methyl transferase kit (CellScript, Madison, WI). 5 μg (229 pmoles) of m^7^G_0_-67mer RNA prepared as described previously was incubated with 25 μmoles SAM and 13 pmoles ^14^C-SAM in ScriptCap reactions prepared according to manufacturer’s instructions. A sham reaction was included containing all reagents except 2‘-O-Methyl transferase enzyme. Reactions were incubated at 37°C for 1 h and then passed through G-25 Sepharose columns twice to remove unreacted SAM. 10 μL of complete reaction mixtures were spotted on filter papers and ^14^C incorporation was measured by liquid scintillation counting. (A) ^14^C incorporation in ScriptCap methyltransferase reactions was measured by scintillation counting in parallel with background samples and sham samples; (B) A ^14^C-SAM standard curve was used to calculate the amount of ^14^C in samples (μCi); (C) Specific activity of the ^14^C-SAM mixture allowed calculation of reaction efficiency by average pmoles of the trace labeled ^14^C incorporated per pmol RNA.
(PPTX)

S2 Fig De novo vs. primed transcription of miniHA. Gel images show de novo synthesis (left lane) and primed RNA synthesis (right lane). RdRp was pre-incubated for 5 minutes in a buffer containing 50 mM Tris-HCl (pH 8.0), 2 mM DTT, 5 mM magnesium acetate, 0.25 U/μL RNAsin, 1.6 μM miniHA template in the presence and absence of 300 μM ApG (Trilink Biotechnologies). Reactions were initiated by addition of NTP substrate mixture containing 0.01 μM α-^33^P-GTP, 1 μM GTP, and 100 μM for each of the rest of NTPs: ATP, CTP and UTP (PerkinElmer, Shelton, CT). To visualize products, aliquots of the reactions were quenched with equal volumes of gel loading dye containing 90% formamide, 100 mM EDTA, 0.1% (w/v) bromphenol blue and xylene cyanol. Products were separated by electrophoresis (15% polyacrylamide, 8 M urea). The dried gels were exposed to phosphorimager screen and visualized using the Typhoon Trio and ImageQuant Software (GE, Piscataway, NJ.)
(PPTX)

S3 Fig cRNP mechanism of action study of guanosine analogs. The mechanism of action of guanosine analogs was interrogated in assays utilizing concentrated cRNP and ^33^P-radiolabeled capped primer with endogenous viral template. Lanes 1–4 show the cleaved RNA primer plus next incoming CTP coded by the endogenous viral RNA, and the product formation in the presence of 2–4 natural NTPs. Lanes 5 and 6 show incorporation of T1106-TP at the first GTP coded by the template sequence, and ablation of long product formation in wells at the top of the gel in the presence of natural nucleotides. Lanes 7 and 8 show a weak band corresponding to incorporation of 2’-FdGTP, and generation of long products in the presence of natural NTP. Lanes 9–14 show neither incorporation of the 4’substituted analogs and T-705-RTP nor formation of long products in the presence of natural NTPs, leaving the MOA of these analogs ambiguous. For this experiment, concentrated cRNP (10% assay volume) was incubated with 460 nM ^33^P-labeled m^7^G_1_-67 for 3 h in buffer containing 100 mM Tris (pH 8.0,) 100 mM KCl, 5 mM MgCl_2_, 1 mM DTT, 0.25% Triton N-101, 10% glycerol, and 0.4 U/μL RNAsin. After 3 h incubation, reactions were quenched with endonuclease inhibitor and 500 μM natural NTPs and/or analogs were added. After 60 minutes, primer extension reactions were quenched with addition of equal volumes of 100 mM EDTA in loading dye. Products were separated by 25% PAGE on a large format gel and quantified by autoradiography. While incorporation and chain termination is observed for T-1106 Triphosphate and stable incorporation is observed for 2’FdGTP, the MOA of the less efficiently incorporated analogs is not discernable.
(PPTX)
