# Supplementary figures and images for: Biochemical characterization of recombinant influenza A polymerase heterotrimer complex: Polymerase activity and mechanisms of action of nucleotide analogs
Source: PLoS One. 2017 Oct 11;12(10):e0185998. doi: 10.1371/journal.pone.0185998 (PMC5636120; doi:10.1371/journal.pone.0185998)

## Slide 1
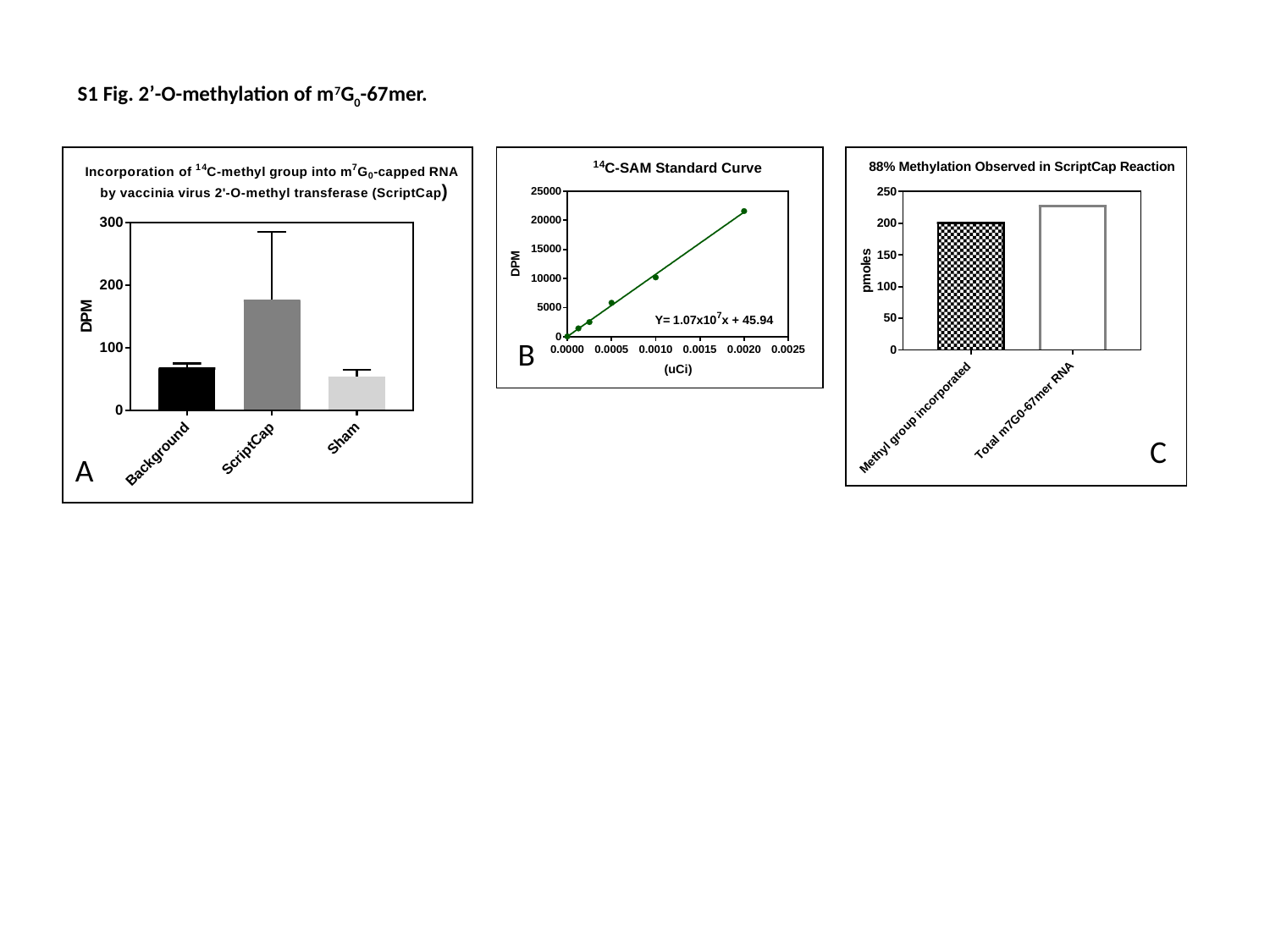

S1 Fig. 2’-O-methylation of m7G0-67mer.
B
C
A

Supplement: S1 Fig — Methylation of m7G0-67mer RNA was assessed by incorporation of 14C from trace labeled S-adenosyl methionine (SAM), (PerkinElmer, Walthham, MA) using the ScriptCap vaccinia virus 2‘-O-Methyl transferase kit (CellScript, Madison, WI). 5 μg (229 pmoles) of m7G0-67mer RNA prepared as described previously was incubated with 25 μmoles SAM and 13 pmoles 14C-SAM in ScriptCap reactions prepared according to manufacturer’s instructions. A sham reaction was included containing all reagents except 2‘-O-Methyl transferase enzyme. Reactions were incubated at 37°C for 1 h and then passed through G-25 Sepharose columns twice to remove unreacted SAM. 10 μL of complete reaction mixtures were spotted on filter papers and 14C incorporation was measured by liquid scintillation counting. (A) 14C incorporation in ScriptCap methyltransferase reactions was measured by scintillation counting in parallel with background samples and sham samples; (B) A 14C-SAM standard curve was used to calculate the amount of 14C in samples (μCi); (C) Specific activity of the 14C-SAM mixture allowed calculation of reaction efficiency by average pmoles of the trace labeled 14C incorporated per pmol RNA. (PPTX) [file pone.0185998.s001.pptx]

## Slide 1
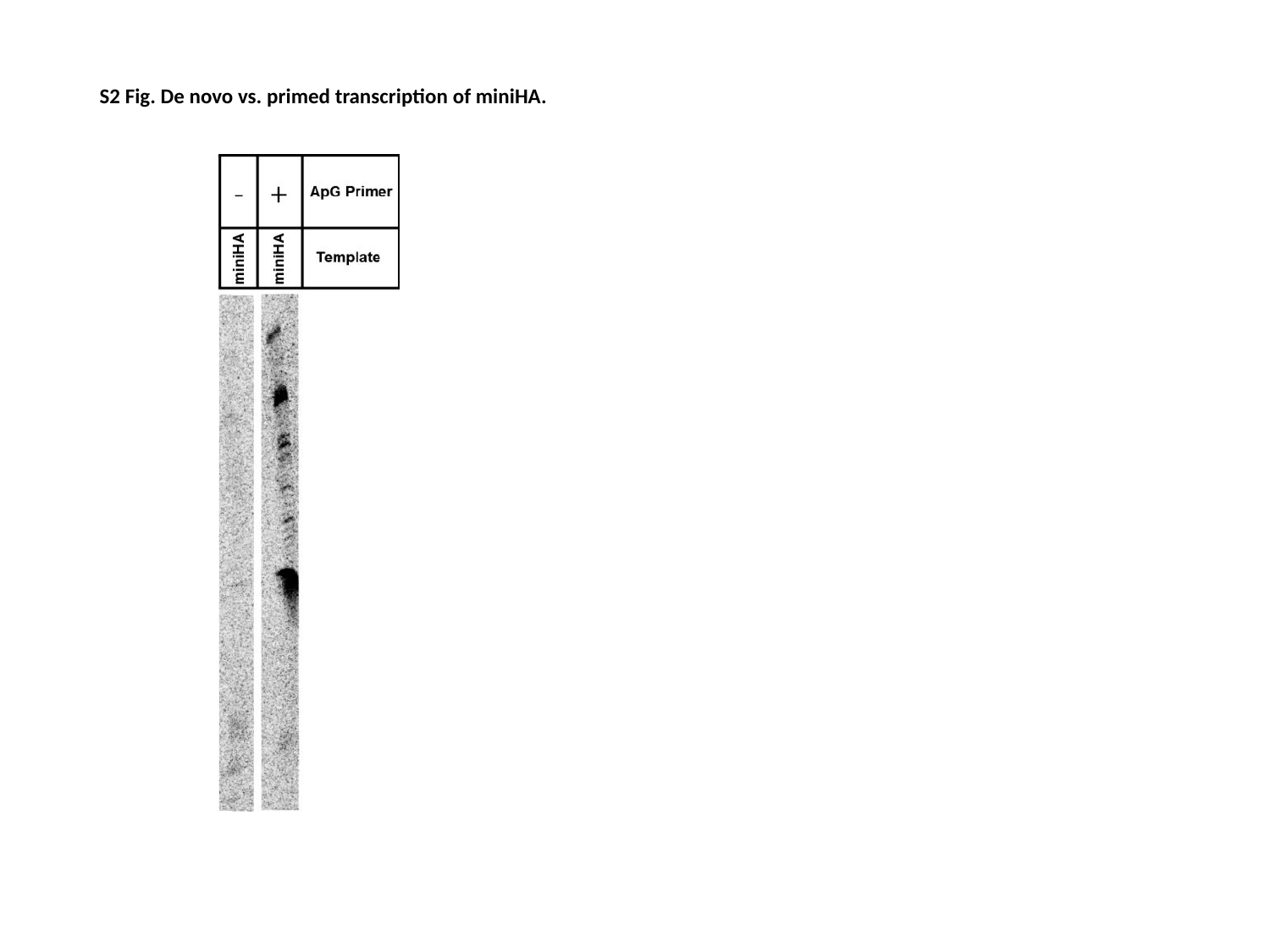

S2 Fig. De novo vs. primed transcription of miniHA.

Supplement: S2 Fig — Gel images show de novo synthesis (left lane) and primed RNA synthesis (right lane). RdRp was pre-incubated for 5 minutes in a buffer containing 50 mM Tris-HCl (pH 8.0), 2 mM DTT, 5 mM magnesium acetate, 0.25 U/μL RNAsin, 1.6 μM miniHA template in the presence and absence of 300 μM ApG (Trilink Biotechnologies). Reactions were initiated by addition of NTP substrate mixture containing 0.01 μM α-33P-GTP, 1 μM GTP, and 100 μM for each of the rest of NTPs: ATP, CTP and UTP (PerkinElmer, Shelton, CT). To visualize products, aliquots of the reactions were quenched with equal volumes of gel loading dye containing 90% formamide, 100 mM EDTA, 0.1% (w/v) bromphenol blue and xylene cyanol. Products were separated by electrophoresis (15% polyacrylamide, 8 M urea). The dried gels were exposed to phosphorimager screen and visualized using the Typhoon Trio and ImageQuant Software (GE, Piscataway, NJ.) (PPTX) [file pone.0185998.s002.pptx]
