# Supplementary material for: Biochemical characterization of recombinant influenza A polymerase heterotrimer complex: Polymerase activity and mechanisms of action of nucleotide analogs
Source: PLoS One. 2017 Oct 11;12(10):e0185998. doi: 10.1371/journal.pone.0185998 (PMC5636120; doi:10.1371/journal.pone.0185998)
Supplement: S3 Fig — The mechanism of action of guanosine analogs was interrogated in assays utilizing concentrated cRNP and 33P-radiolabeled capped primer with endogenous viral template. Lanes 1–4 show the cleaved RNA primer plus next incoming CTP coded by the endogenous viral RNA, and the product formation in the presence of 2–4 natural NTPs. Lanes 5 and 6 show incorporation of T1106-TP at the first GTP coded by the template sequence, and ablation of long product formation in wells at the top of the gel in the presence of natural nucleotides. Lanes 7 and 8 show a weak band corresponding to incorporation of 2’-FdGTP, and generation of long products in the presence of natural NTP. Lanes 9–14 show neither incorporation of the 4’substituted analogs and T-705-RTP nor formation of long products in the presence of natural NTPs, leaving the MOA of these analogs ambiguous. For this experiment, concentrated cRNP (10% assay volume) was incubated with 460 nM 33P-labeled m7G1-67 for 3 h in buffer containing 100 mM Tris (pH 8.0,) 100 mM KCl, 5 mM MgCl2, 1 mM DTT, 0.25% Triton N-101, 10% glycerol, and 0.4 U/μL RNAsin. After 3 h incubation, reactions were quenched with endonuclease inhibitor and 500 μM natural NTPs and/or analogs were added. After 60 minutes, primer extension reactions were quenched with addition of equal volumes of 100 mM EDTA in loading dye. Products were separated by 25% PAGE on a large format gel and quantified by autoradiography. While incorporation and chain termination is observed for T-1106 Triphosphate and stable incorporation is observed for 2’FdGTP, the MOA of the less efficiently incorporated analogs is not discernable. (PPTX) [file pone.0185998.s003.pptx]

## Slide 1
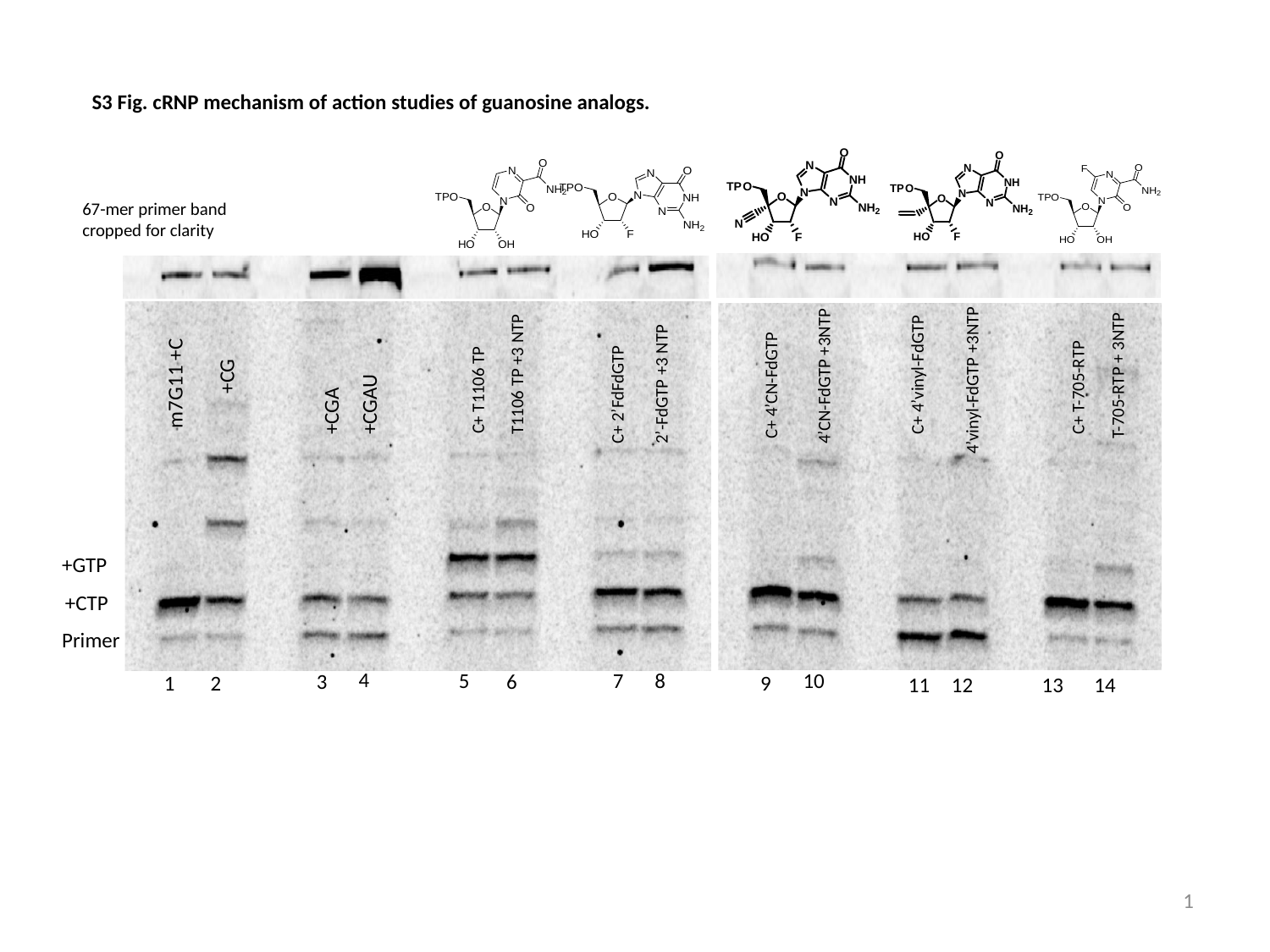

S3 Fig. cRNP mechanism of action studies of guanosine analogs.
67-mer primer band
cropped for clarity
+CG
T1106 TP +3 NTP
2’-FdGTP +3 NTP
m7G11 +C
+CGA
+CGAU
C+ T1106 TP
C+ 4’vinyl-FdGTP
C+ T-705-RTP
T-705-RTP + 3NTP
C+ 4’CN-FdGTP
C+ 2’FdFdGTP
4’CN-FdGTP +3NTP
4’vinyl-FdGTP +3NTP
+GTP
+CTP
Primer
4
10
5
7
8
3
6
1
2
9
11
12
13
14
1
